# Combined expectancies: electrophysiological evidence for the adjustment of expectancy effects

**DOI:** 10.1186/1471-2202-7-37

**Published:** 2006-05-04

**Authors:** Uwe Mattler, Arie van der Lugt, Thomas F Münte

**Affiliations:** 1 Department of Neurology II, Center for Advanced Imaging, Otto-von-Guericke-University Magdeburg, Leipziger Strasse 44 (Haus 1), D-39120 Magdeburg, Germany; 2 Maastricht University, Faculty of Psychology, Department of Cognitive Neuroscience, P.O. Box 616, 6200 MD Maastricht, The Netherlands; 3 Department of Neuropsychology, Institute of Psychology II, Otto-von-Guericke University Magdeburg, P.O. Box 4120, D-39016 Magdeburg, Germany

## Abstract

**Background:**

When subjects use cues to prepare for a likely stimulus or a likely response, reaction times are facilitated by valid cues but prolonged by invalid cues. In studies on combined expectancy effects, two cues can independently give information regarding two dimensions of the forthcoming task. In certain situations, cueing effects on one dimension are reduced when the cue on the other dimension is invalid. According to the Adjusted Expectancy Model, cues affect different processing levels and a mechanism is presumed which is sensitive to the validity of early level cues and leads to online adjustment of expectancy effects at later levels. To examine the predictions of this model cueing of stimulus modality was combined with response cueing.

**Results:**

Behavioral measures showed the interaction of cueing effects. Electrophysiological measures of the lateralized readiness potential (LRP) and the N200 amplitude confirmed the predictions of the model. The LRP showed larger effects of response cues on response activation when modality cues were valid rather than invalid. N200 amplitude was largest with valid modality cues and invalid response cues, medium with invalid modality cues, and smallest with two valid cues.

**Conclusion:**

Findings support the view that the validity of early level expectancies modulates the effects of late level expectancies, which included response activation and response conflict in the present study.

## Background

Ready, steady, go! As suggested by everyday experience and substantiated by numerous studies, performance can be improved when events are expected. Research on selective attention has examined expectancy effects regarding various perceptual dimensions of the forthcoming task. For instance, in a typical modality cueing task, advance knowledge of the likely modality of the target stimulus is given by a pre-cue [1,2]. The common finding is that on valid trials, in which the target appears in the expected modality, reaction time (RT) is reduced and response accuracy is increased. In contrast, on invalid trials, in which the target appears in an unexpected modality, RT is prolonged and accuracy is reduced [1,2]. Similar effects of pre-cues have been demonstrated for cues regarding stimulus location [3], stimulus color [4], form [5], or features [6]. Beyond the perceptual domain, motor responses have also been found to be affected by expectancies regarding the likely motor response [7,8]. Advance knowledge about the likely motor response improves performance on valid trials, but it impairs performance on invalid trials. Although most laboratory studies have examined the effects of a single particular expectancy, it is far more common in everyday situations that we develop a set of expectancies regarding several dimensions of the forthcoming situation. For example, one can conceive of driving to a stop light that turns from green to yellow as involving the concurrent expectancy to see a red light and the expectancy to hit the brakes. To investigate how different expectancies act together, a number of studies have begun to examine the effects of combined expectancies on performance measures (for a review see [2]). The present study extends this research by assessing expectancy effects with electrophysiological measures.

Early studies have already revealed that expectancy effects are sometimes reduced when combined with other expectancies [9-14]. For instance, Klein and colleagues [11,12,15] combined spatial cueing with advance knowledge of the target stimulus by presenting one target more frequently than its alternative. Normal spatial cueing effects were observed for the frequent stimulus, with faster responses on valid trials and slower responses on invalid trials. However, spatial cueing effects were absent on trials with the infrequent stimulus: RT was about as long on valid as on invalid trials. Recent research revealed that combined expectancies interact regardless of what specific expectancies are combined, including the combinations of two stimulus-related expectancies [9,16], a stimulus-related expectancy and a response-related expectancy [2], or two response-related expectancies [16]. These findings suggest that *expectancy interaction *results from a rather general mechanism that operates in many situations in which multiple expectancies act together [2,16].

One of the first accounts of combined expectancy effects explained the elimination of spatial cueing effects in conditions with unexpected response requirements in terms of the *spotlight failure hypothesis *[11]. According to spotlight failure, visual attention does not always improve early visual processing, contrary to the hypothesis that attention operates like a spotlight [3]. However, results of Klein and Hansen [11] are also consistent with the alternative *spotlight masking *perspective, which proposes that spatial cueing effects on early perceptual processing do not appear in performance measures because of later processes which are concerned with response processing [12]. This view has been supported by a recent electrophysiological study by Handy, Green, Klein and Mangun [17] who found that early visual potentials were modulated by spatial cueing but not by stimulus-response related expectancies. A similar view was put forward by Kingstone and Klein [10] and Kingstone [9], who focused on the interaction between expectancies regarding different perceptual dimensions of a target stimulus (e.g., form and location). According to Kingstone's [9]*crosstalk hypothesis*, the attentional commitment to the possibility that an expectancy will be confirmed depends on the confirmation/disconfirmation of expectancies regarding other perceptual dimensions.

**Figure 1:**
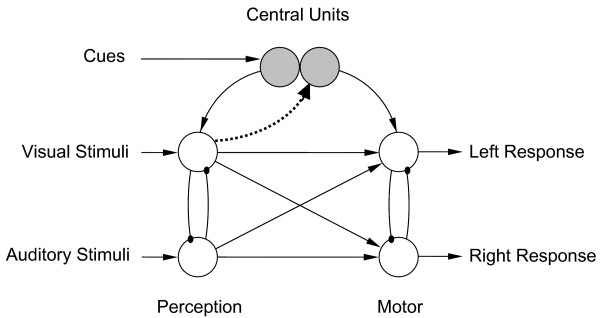
**Illustration of the architecture of the Adjusted Expectancy Model**. Two identical stages, each consisting of two competitive units of alternative representations, are connected in series. Cues are interpreted in central units leading to increased activation of the corresponding representation at perceptual and motor stages of processing. Target stimuli activate their representations at the perceptual stage. When the target activates the representation that was activated by the cue, central units are informed (dotted line) to provide additional activation for the cued representations at the motor stage. When the target and the cue activate different representations at the perceptual stage, central units are informed to reduce the additional activation of the cued representation at the motor stage. Expectancy interaction occurs because the evaluation of perceptual expectancies modulates post-target activation of motor representations. Note, excitatory links end with an arrow, and inhibitory links end with a dot. Only examples of essential links are included to illustrate the basic principles of the model (see [18]).

To cover the entire range of expectancy interactions, Mattler [2,16,18] extended and specified the crosstalk hypothesis in terms of the Adjusted Expectancy Model (Figure 1). According to this model, there are two effects of expectancies. One is realized prior to target presentation by activating those representations which are related to the processing of the expected event. The other consequence of an expectancy is additional activation of these representations which takes place during target processing. This post-target effect of expectancies can be adjusted corresponding to the confirmation or disconfirmation of other expectancies. More specifically, consider an experiment in which subjects have to respond to a red light or a low pitch tone with a left hand response, and to a green light or a high pitch tone with a right hand response. Prior to target presentation, subjects get a double cue that tells them the likely target stimulus and the likely response. In this case the model assumes that subjects prepare for the cued stimulus modality and the cued response prior to target presentation. After target presentation, the system evaluates the stimulus modality and finds that either the modality of the target corresponds to the expected modality or not. In the case of a confirmed modality expectation, central instances allow further activation of the expected response. This post-target activation leads to facilitated response processing when the response cue is valid, but increased processing difficulties when the response cue is invalid. If target processing reveals that the modality expectation is invalid, however, this result is signaled to central instances which reduce the post-target activation of the expected response. Consequently, when the response cue is valid, the reduced post-target activation of the expected response has the effect that the facilitation of response processing is also reduced. In the case of an invalid response cue, however, reduced post-target response activation leads to reduced difficulties in response processing, resulting in relatively fast and accurate responses [18].

**Figure 2:**
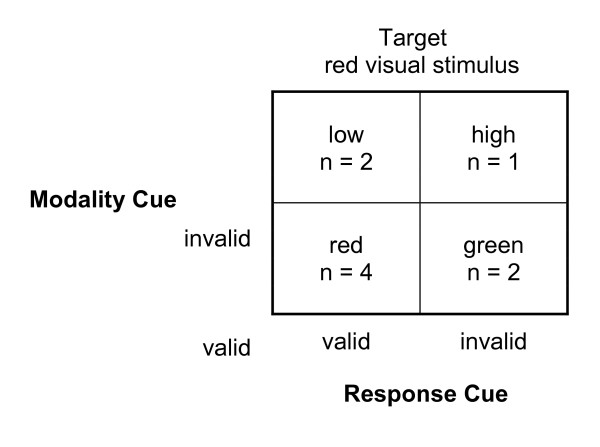
**Combination of modality cueing and response cueing by word cues**. In each trial, a simple word was presented as a cue carrying two pieces of information: the likely stimulus modality of the target, and the likely response required. For each target stimulus four possible combinations of valid and invalid information regarding stimulus modality and response occurred with unequal frequencies. Word cues are given here for the example of a red target stimulus which required a left hand response. n = frequency of each combination. English translations of the German words are provided.

**Figure 3:**
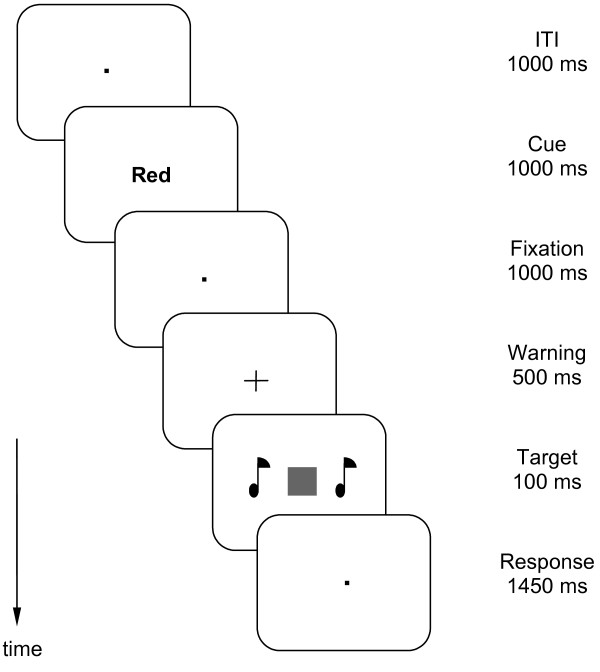
Schematic diagram of the sequence of events in the experiment

The study on hand presents a test of the Adjusted Expectancy Model. To this end, we combined modality cueing with response cueing and collected both behavioral and electrophysiological measures. In line with previous experiments [2], we used single words that announced the forthcoming stimulus and the required response. Each cue was valid on two out of three trials (Figure 2). The sequence of events is depicted in Figure 3.

### Predictions regarding the lateralized readiness potential (LRP)

The time-course of motor activation was assessed by measuring the lateralized readiness potential (LRP). The LRP is derived by averaging the hemispheric asymmetry in event related brain potentials that is obtained in left-hand responses and right-hand responses over the primary motor cortex (for details see [19] and the *Methods *section). The LRP reflects the relative activation of motor cortical areas of the responding hand. It provides a real-time index of selective response activation (for reviews see [20,21]) and has been used to measure the effects of response cues [22]. According to the *Adjusted Expectancy Model *there are two phases of cue-related response activation, before and after target presentation. Because post target activation depends on the validity of modality cues, the initial part of the response activation after target presentation as measured by the LRP should be modulated corresponding to the validity of the modality cue: the LRP of post-target activation of the cued response should continue to increase on trials with valid modality cues because early perceptual processing revealed that perceptual expectancies were confirmed; on trials with invalid modality cues, however, the LRP of post-target activation of the cued response should not increase with the same rate because early perceptual processing disconfirmed perceptual expectancies and signaled to central processing units that the rate of post-target response activation should be reduced [18].

### Predictions regarding the N200 component of the ERP

In addition, we were interested in the control processes which are involved in processing unexpected events. To this end, we examined the effects of combined expectancies on the N200, an ERP component that has been interpreted as a marker of control processes that operate in a variety of situations [23,24]. According to the *Adjusted Expectancy Model*, response activation is increased on trials with valid modality cues due to the confirmation of the perceptual expectancy. This leads to increased activation of the incorrect response on trials with valid modality cues and invalid response cues. In these trials the processing of a correct response should lead to an increased response conflict that should be reflected in a large N200 amplitude [18]. In contrast, the model predicts a much smaller response conflict for trials with two invalid cues, because in these trials early perceptual processing already reveals that perceptual expectancies are violated and terminates post-target response activation. Thus, the model predicts that the N200 amplitude is largest on trials with valid modality cues and invalid response cues, intermediate with two invalid cues, but smallest with two valid cues.

The present study focuses on the electrophysiological responses that are related to expectancy interaction on behavioral measures. Previous research has shown that the extent of expectancy interaction varies between subjects [2,18]. The Adjusted Expectancy Model accounts for inter-individual differences by assuming that the extent of expectancy interaction depends on whether the expectancies are related in the subject's internal representations of them. The establishment of an internal relationship between expectancies might be induced by explicit instructions, derived implicitly, or generated by subjects because of prior experience or individual dispositions. One way to modulate the interaction between expectancy effects is to use separated or integrated cues [2,16]: When expectancies are induced by integrated cues, which may consist of two physically connected visual features, expectancy effects frequently interact. In contrast, when cues consist of two separable parts, like a word and an arrow, expectancy effects remain largely independent. The present experiment used integrated cues to induce expectancy interaction. Because this study focuses on the physiological responses to expectancy interaction, only those subjects that showed expectancy interaction on behavioral measures were included in the present study (see Methods).

## Results

### Behavioral results

#### RT

**Figure 4:**
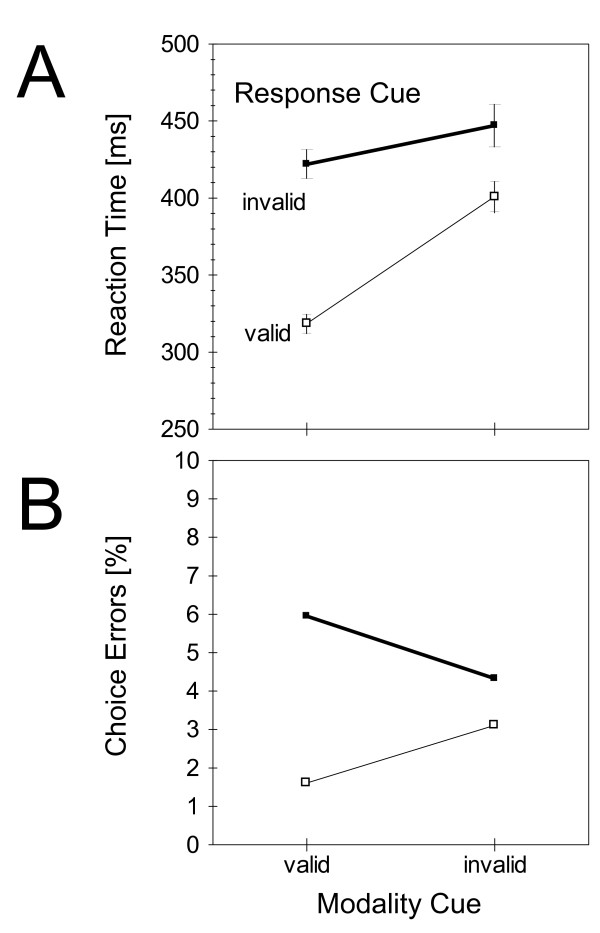
**Performance measures as a function of the combination of modality cueing and response cueing**. **A**, reaction time. Error bars show twice the mean standard error of RTs of single trials for each condition, calculated for each subject and then averaged across subjects. **B**, choice error rates.

RTs were shorter (359 msec) to valid response cues than to invalid response cues (434 msec; F(1, 17) = 152.9, p < .001). The validity of the modality cue also had a significant effect on RT, with 370 and 424 msec for valid and invalid cues, respectively (F(1, 17) = 28.3, p < .001). The interaction of modality cueing and response cueing was significant (F(1, 17) = 93.3, p < .001). As Figure 4A shows, the effect of response cues was reduced in trials with invalid modality cues (47 msec) as compared to trials with valid modality cues (103 msec). Follow-up tests (bonferroni-corrected critical p-value = .0083) revealed that RT was shortest with two valid cues, when compared to invalid modality cues and valid response cues (F(1, 17) = 44.5, p < .001), when compared to valid modality cues and invalid response cues (F(1, 17) = 228.6, p < .001), and also when compared to two invalid cues (F(1, 17) = 85.0, p < .001). RT was shorter with valid modality cues and invalid response cues than with two invalid cues (F(1, 17) = 9.4, p = .007) but not significantly longer than with invalid modality cues and valid response cues (F(1, 17) = 5.2, p = .036). Finally, in conditions with invalid modality cues, RT was shorter with valid than with invalid response cues (F(1, 17) = 49.9, p < .001).

#### Errors

Errors occurred on 3.8% of the trials. Mean error rates were significantly affected by the validity of the response cue, with means of 2.4% and 5.1% for valid and invalid response cues, respectively (F(1, 17) = 30.0, p < .001). The validity of the modality cue had no significant effect on error rates (F(1, 17) < 1, p = .38). However, the interaction of modality cue and response cue was significant (F(1, 17) = 34.1, p < .001), indicating that the effect of the response cue was reduced in trials with invalid modality cues (1.2 %) as compared to trials with valid modality cues (4.3 %). Figure 4B shows mean error rates for each condition. Follow-up tests (bonferroni-corrected critical p-value = .0083) revealed that responses were most accurate with two valid cues, when compared to invalid modality cues and valid response cues (F(1, 17) = 23.1, p < .001), when compared to valid modality cues and invalid response cues (F(1, 17) = 53.1, p < .001), and also when compared to two invalid cues (F(1, 17) = 30.0, p < .001). Error rates in the condition with valid modality cues and invalid response cues were marginally larger than in the condition with invalid modality cues and valid response cues (F(1, 17) = 8.8, p = .009), and also only marginally larger than in the condition with two invalid cues (F(1, 17) = 4.1, p = .059). Finally, with invalid modality cues, error rates did not differ significantly between conditions with valid and invalid response cues (F(1, 17) = 3.4, p = .083).

### ERP results

#### LRP

**Figure 5:**
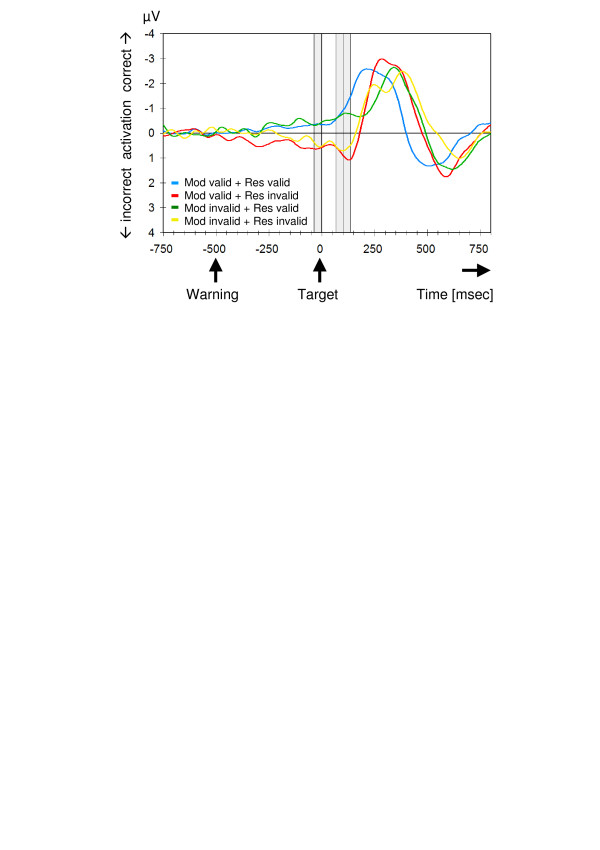
**Stimulus-locked lateralized readiness potentials as a function of the combination of modality cueing and response cueing**. Valid modality cues and valid response cues (blue line), valid modality cues and invalid response cues (red line), invalid modality cues and valid response cues (green line), invalid modality cues and invalid response cues (yellow line). At t = 0 the target stimulus was presented. The baseline for this analysis consisted in the activity in the interval -748 to -648 ms before target presentation. The three grey fields mark the time intervals over which the cueing effects on the LRP were analyzed.

Figure 5 shows the grand average of stimulus-locked LRPs for each of the four conditions beginning 750 ms before target onset. Visual inspection of stimulus-locked LRPs shows that *prior to target onset *the LRP was biased by response cue validity: on trials with valid response cues the LRP was biased towards negative values which corresponds to an activation of the correct response hand, whereas trials with invalid response cues produced positive values in the LRP, corresponding to increased activation of the incorrect response hand. *After target onset*, these effects of response cues were modulated by the validity of modality cues. Response cueing effects on the LRP were increased on trials with valid modality cues, but decreased on trials with invalid modality cues, both with valid and with invalid response cues.

**Figure 6:**
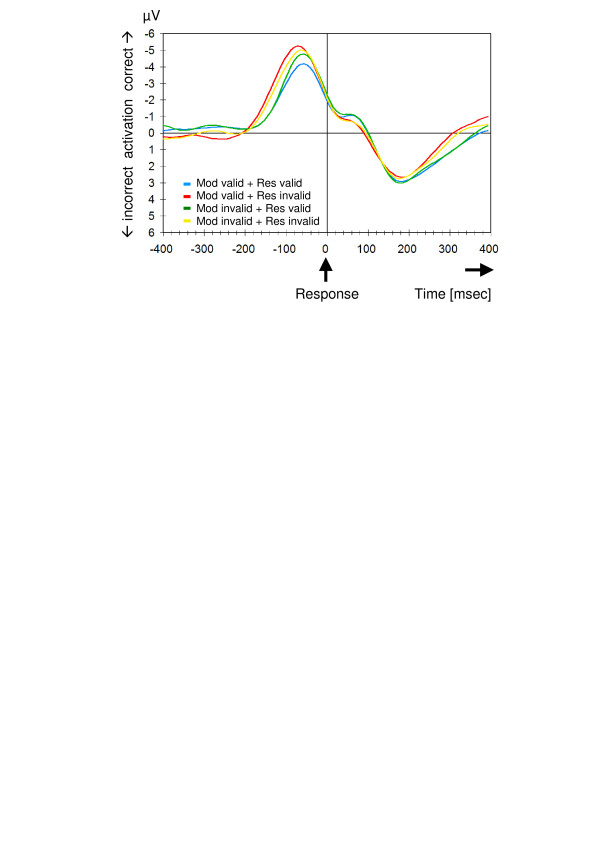
**Response-locked lateralized readiness potentials as a function of the combination of modality cueing and response cueing**. At t = 0 the response was measured with the response key. The baseline for this analysis consisted in the activity in the interval -1648 to -1548 ms before the response.

Figure 6 shows the grand average of response-locked LRPs for each of the four conditions. Visual inspection shows that the temporal interval from the onset of correct response-locked LRPs to the point in time when the response key was closed (t = 0) was longer on trials with invalid as compared to trials with valid response cues.

These observations were corroborated by statistical analyses. First, on trials with *invalid response cues*, the LRP reached positive values that differed significantly from zero prior to target presentation in the time interval -32 to 0 msec, both with valid (t(17) = 3.6, p < .01, two-sided) and invalid modality cues (t(17) = 2.4, p < .03, two-sided). After target onset, these response cueing effects were still observed in the time interval 68 to 100 msec after target onset with valid (t(17) = 4.0, p < .01, two-sided) and invalid modality cues (t(17) = 3.1, p < .01, two-sided). In both intervals the LRPs in conditions with valid and invalid modality cues did not differ significantly (t(17) = 1.2, p = .24, two-sided; t(17) = 0.3, p = .78, two-sided; respectively). In the time interval 108 to 140 msec after target onset, the LRP maintained significantly above zero with valid (t(17) = 7.5, p < .01, two-sided) and invalid modality cues (t(17) = 2.7, p < .02, two-sided). Most importantly, corresponding to the prediction of the model this incorrect activation increased significantly from the interval 68–100 msec to the interval 108–140 msec after target onset when modality cues were valid (t(17) = 2.0, p = .03, one-sided) but not when modality cues were invalid (t(17) = 0.6, p = .58, two-sided). As a consequence, in the interval 108 to 140 msec after target presentation the LRP was significantly more positive with valid compared to invalid modality cues (t(17) = 2.0, p = .03, one-sided).

Second, on trials with *valid response cues*, the LRP reached negative values that differed significantly from zero in the time interval -32 to 0 msec before target onset with valid and invalid modality cues (t(17) = -3.4, p < .01, and t(17) = -2.9, p < .01, respectively, two-sided), and also in the time interval 68 to 100 msec after target onset (t(17) = -6.3, p < .001, and t(17) = -3.9, p = .001, respectively, two-sided). In both intervals the LRPs in conditions with valid and invalid modality cues did not differ significantly (t(17) = -0.1, p = .92, two-sided; t(17) = -0.3, p = .74, two-sided). Corresponding the predictions, the correct (negative) activity increased significantly with valid modality cues between the interval 68 to 100 msec and the interval 108 to 140 msec (t(17) = 4.1, p = .001, two-sided) but not with invalid modality cues (t(17) = 0.7, p = .48, two-sided). However, 108 to 140 msec after target presentation the correct activity remained significantly below zero with invalid modality cues (t(17) = -3.8, p = .001, two-sided). Moreover, in the interval 108 to 140 msec after target onset the correct activity increased with valid as compared to invalid modality cues (t(17) = -2.6, p = .021, two-sided). Note that the same pattern of findings is obtained when we eliminate the response cue induced offset at the point of target presentation.

#### Onset latencies of stimulus- and response-locked LRPs

**Figure 7:**
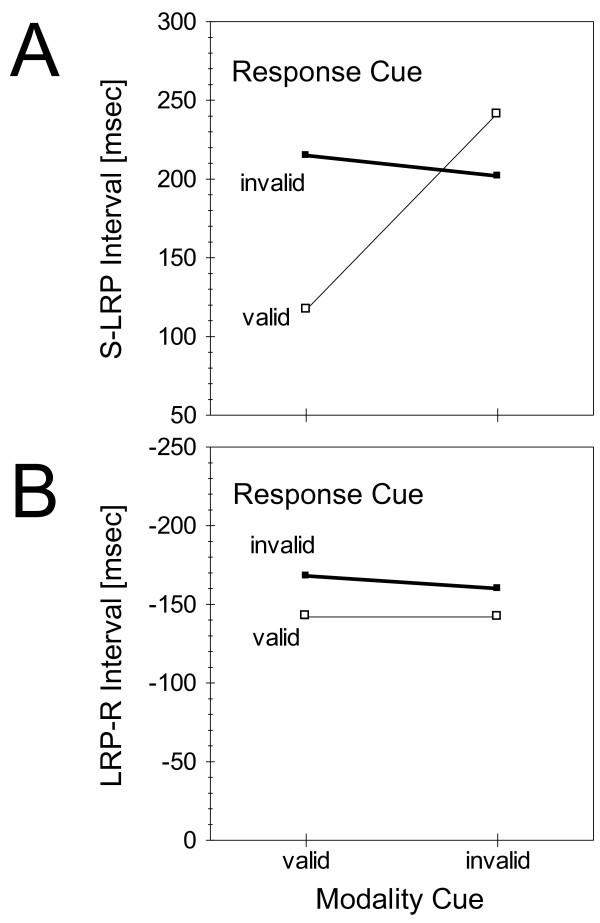
**LRP onset latencies as a function of the combination of modality cueing and response cueing**. **A**, onset latency of correct stimulus-locked LRPs (S-LRP). **B**, onset latency of response-locked LRPs (LRP-R).

Statistical analysis of the onset latency of correct stimulus-locked LRPs revealed a significant main effect of modality cueing, with 166 and 222 msec for valid and invalid modality cues, respectively (F_c_(1, 17) = 25.9, p < .001). The interaction of modality cueing and response cueing was also significant (F_c_(1, 17) = 18.4, p < .001). Figure 7A shows a large effect of response cues of 98 msec on trials with valid modality cues (F_c_(1, 17) = 31.0, p < .001), and a reversed response cueing effect of -40 msec in trials with invalid modality cues which did not reach significance (F_c_(1, 17) = 1.9, p = .19). Stimulus locked LRP onset latency was slightly earlier on trials with invalid modality cues and invalid response cues (202 msec) than with valid modality cues and invalid response cues (215 msec), however, this effect did not reach significance (F_c_(1, 17) < 1, p = .44).

Statistical analysis of the onset latencies of response-locked LPRs revealed a marginally significant effect of response cue validity (F_c_(1, 17) = 3.8, p = .069). Mean LRP-R intervals were shorter for trials with validly (-142 msec) as compared to invalidly cued responses (-164 msec). No other effect reached significance with any threshold (F_c _< 2.7, p > .12 in all other cases). Figure 7B shows the mean LRP-R intervals for each condition.

#### N200

**Figure 8:**
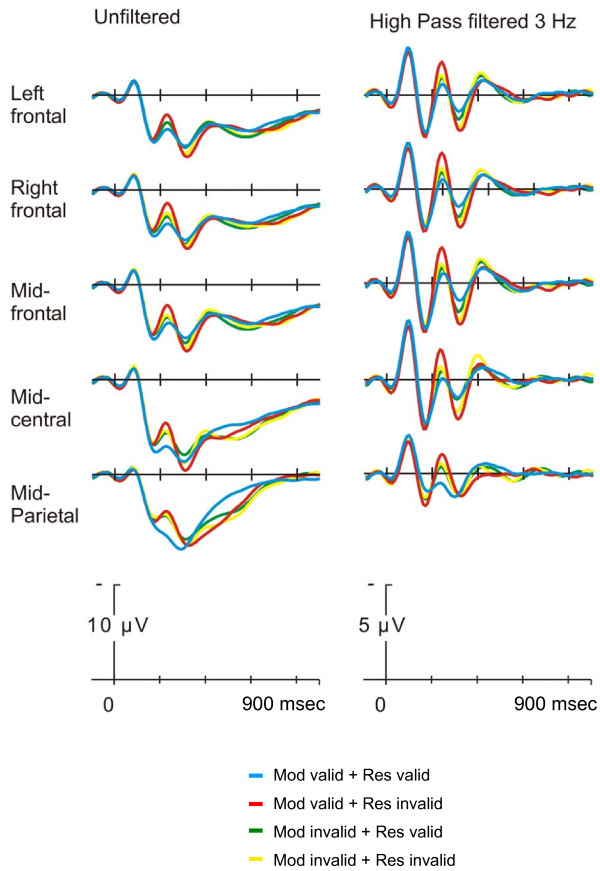
**Stimulus-locked grand average waveforms as a function of the combination of modality cueing and response cueing**. *Left column*, unfiltered grand averages at frontal and midline electrodes. *Right column*, 3 Hz high-pass filtered grand averages. At t = 0 the target stimulus was presented.

**Figure 9:**
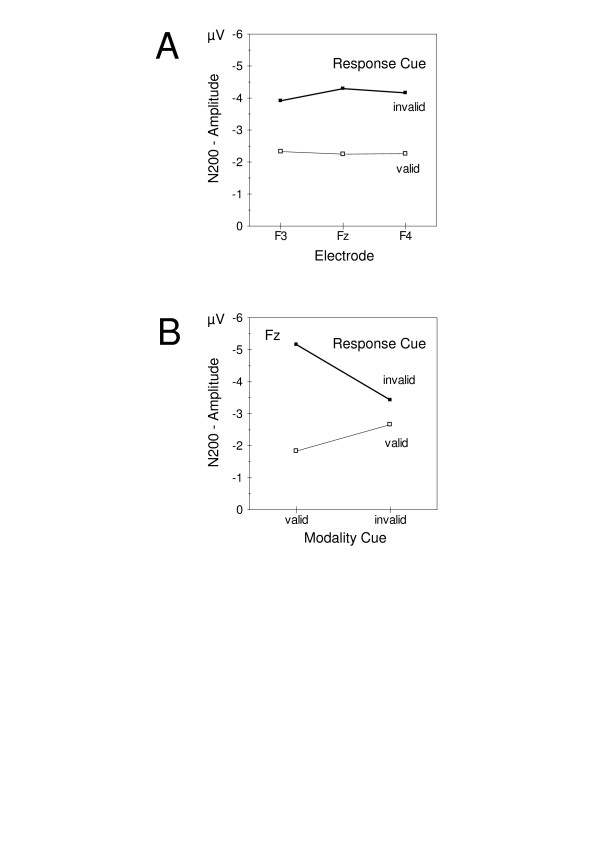
**Effects on N200 amplitude**. **A**, effects of response cues on the N200 peak amplitude at the three frontal electrode positions. **B**, cueing effects on N200 peak amplitude at electrode Fz as a function of modality cueing and response cueing.

The left column of Figure 8 shows the grand average waveforms of the three frontal electrodes and the midline electrodes for each of the four conditions. Because the N200 was superimposed on a large positive wave, we examined the N200 after the application of a 3 Hz high-pass filter (right column of Figure 8). The ERPs at frontal sites were characterized by an initial negativity at about 80 msec followed by a steep positive deflection peaking at about 170 msec and a further negative wave with a maximum at about 230 msec (N200). The N200 showed clear cue related effects that were smallest for validly cued targets and largest for targets preceded by a valid modality cue but invalid response cue. The amplitude of the N200 was defined as the amplitude of the largest peak in the time interval between 160 and 320 msec after target onset at the three frontal electrode positions (F3, Fz and F4). The statistical analysis revealed a significant effect of response cue validity (F(1, 13) = 54.7, p < .001) with means of -2.3 μV and -4.1 μV for valid and invalid response cues, respectively. The effect of response cue validity differed across electrode positions (F(2, 26) = 4.9, p = .028). Separate tests revealed that response cueing effects were significant at each of the three electrode positions (F(1, 13) > 45, p < .001 in all cases). The response cueing effect – determined as the difference between N200 amplitude on trials with invalid and valid response cues – was significantly smaller at F3 than at Fz (t(13) = 4.2, p = .001) with means of -1.6 μV and -2.1 μV, respectively (see Figure 9A).

Of most importance, however, is the significant interaction between modality cueing and response cueing (F(1, 13) = 60.8, p < .001). This interaction did not vary with electrode position (F(2, 26) < 1, p = .77). Therefore, the following analysis focused on electrode Fz. Figure 9B shows that the N200 amplitude was modulated by response cueing to a greater extent when modality cues were valid than when they were invalid. The largest N200 peak amplitude was elicited in conditions with valid modality cues and invalid response cues, and the smallest N200 amplitude occurred in conditions with two valid cues. This was confirmed by statistical analyses of the amplitudes in the different conditions. The N200 amplitude was increased in conditions with valid modality cues and invalid response cues relative to the condition with two valid cues (F(1, 13) = 83.3, p < .001), the condition with invalid modality and valid response cues (F(1, 13) = 50.4, p < .001), and relative to the condition with two invalid cues (F(1, 13) = 17.7, p = .001). The N200 amplitude was reduced with two valid cues when compared to the condition with invalid modality and valid response cues (F(1, 13) = 8.9, p = .011), and also when compared to the condition with two invalid cues (F(1, 13) = 12.7, p = .004). Finally, the N200 amplitude was larger in the condition with two invalid cues than in the condition with invalid modality and valid response cues (F(1, 13) = 6.4, p = .025). Analyses of the N200 peak latency (filtered and unfiltered) revealed no significant effect of any experimental variable (F < 2.8, p > .1 in all cases).

**Figure 10:**
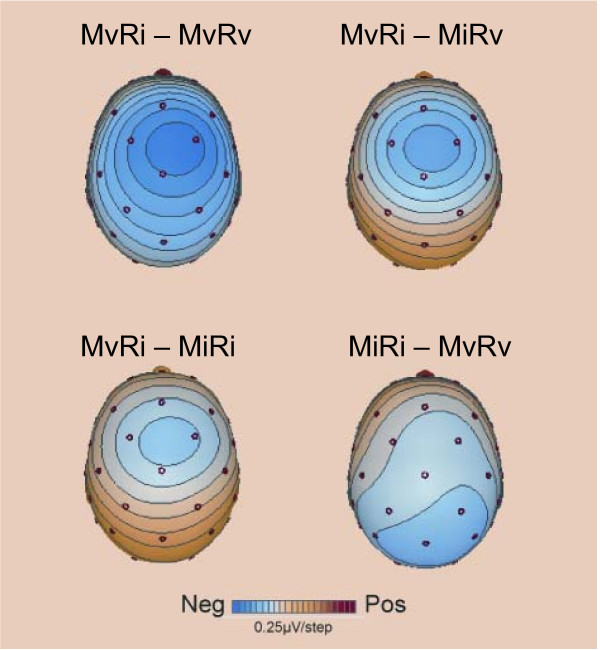
**Topography N200 amplitude**. Topography of N200 peak amplitude 220 ms after target onset based on high-pass filtered data as a function of modality cueing and response cueing. Each map shows a specific difference between two conditions. Mv = valid modality cue, Mi = invalid modality cue, Rv = valid response cue, Ri = invalid response cue.

The topography of the N200 220 ms after target onset in Figure 10 shows two effects. A similar frontocentral negativity was observed in the condition with valid modality cues and invalid response cues, irrespective of which condition was used for comparison. In addition, a parietal negativity was found in the condition with two invalid cues when compared to the condition with two valid cues.

## Discussion

This study employed electrophysiological measures to test predictions derived from the *Adjusted Expectancy Model *which attempts to account for combined expectancy effects [18]. We examined two electrophysiological measures to study neurophysiological effects of the combination of modality cueing and response cueing: the LRP as a real-time index for response activation, and the amplitude of the frontal N200 component as a signature for processes related to conflict processing. The Adjusted Expectancy Model predicts a specific time course of the LRP after target presentation: With valid modality cues, the initial part of the LRP should show an increase of the response cue-related activity; with invalid modality cues, however, the initial part of the LRP should show no such increase of the response cue-related activity. For the N200 amplitude, the model predicts that the effect of response cues is modulated by the validity of modality cues. Therefore, the largest conflict and the largest N200 amplitude was predicted for the case of valid modality cues and invalid response cues, an intermediate amplitude for two invalid cues, and the smallest amplitude for two valid cues.

Behavioral findings are in line with those of previous experiments which showed larger response cueing effects in trials with valid rather than invalid perceptual cues [2,11,12]. Measures of the LRP confirmed the prediction of the Adjusted Expectancy Model by showing that the effect of both valid and invalid response cues on motor activation increased with valid modality cues when compared to the LRP with invalid modality cues. Analyses of the N200 peak amplitude also confirmed the prediction of the model by showing the largest N200 amplitude on trials with valid modality cues and invalid response cues, and a smaller N200 amplitude on trials with two invalid cues. With two valid cues the N200 amplitude was smallest, but it was medium sized with invalid modality cues and valid response cues. These findings support the view that the validity of early level expectancies can affect the processing at later levels via an adjustment of the support of late level expectancy effects [9,10,12] as suggested by the Adjusted Expectancy Model [18].

### How persuasive are the LRP-effects?

In conditions with *valid response cues *the LRP after target onset showed an early lateralization towards the correct hand (Figure 5). The time course of this correct lateralization was modulated by the validity of modality cues. With valid modality cues, correct lateralization increased continuously. With invalid modality cues, however, the lateralization remained at a low level that was nevertheless significantly below zero. These findings correspond to the predictions of the Adjusted Expectancy Model that response related expectancies receive further support after target presentation in trials with valid modality cues, but not in trials with invalid modality cues. However, this time course of the LRP is also consistent with the alternative view that subjects are especially efficient in processing on trials with two valid cues.

The findings from *invalid response cues*, however, provide important support for the Adjusted Expectancy Model but not for accounts that assume prolonged target processing in conditions with invalid modality cues. The time course of the LRP after target onset shows that the incorrect response was activated to a greater degree with valid modality cues than with invalid modality cues. In addition, the latency of correct response activation conflicts with the view that invalid modality cues simply prolong target processing because the latency of correct response activation was not different in trials with valid and invalid modality cues.

The literature points to a limitation of the interpretation of LRP measures which results from the fact that the LRP reflects a mixture of preparation-related and execution-related activations [25]. As one consequence, the activation of the incorrect response in the LRP could result from partially activated responses [19]. In the present case, however, our conclusions would hold even when modality cues affect the proportion of partial response error rates. This is because the model distinguishes between preparatory activation prior to target presentation and additional activation which follows after target presentation. Regarding the later activation it simply predicts that valid modality cues lead to an increase of response activation which should be seen in the LRP amplitude and also in an increased error rate when response cues are invalid. Whether or not incorrect activation triggers an erroneous response depends on response threshold, and the question how preparation- and execution-related activation relate to each other is not addressed by the model. Because we found both, effects in the LRP amplitudes and in the error rates, we conclude that our data support the model.

One attempt to assess the contribution of preparation-related and execution-related effects consists in the separate analysis of stimulus- and response-locked LRPs [22,25,28]. In the present study, analyses of the onset of correct stimulus-locked LRPs revealed that latencies were modulated by the interaction of modality and response cue validity: a large effect of response cue validity in trials with valid modality cues was reversed in trials with invalid modality cues. These effects of modality cueing and response cueing on stimulus-locked LRP latencies do not fit precisely to the pattern of effects observed in behavioral measures of RT. This discrepancy corresponds to findings of neurophysiological and psychophysiological studies of response preparation which report response cueing effects at a motor level which are only weakly related to response speed effects of response cues [26,27]. On the other hand, however, there have been reports of response cueing effects at late levels of the motor system in the spinal cord [26]. To assess the contribution of late level response activation effects we conducted an analysis of response-locked LRP latencies which revealed longer latencies with invalid as compared to valid response cue conditions. Taking these late level effects of response cueing into account, the pattern of expectancy interaction which we observed in behavioral measures (Figure 4) can be approximated by a combination of the more preparation-related effects of modality and response cueing, and the more execution-related effects of response cueing (Figure 7). To sum up, outcomes suggest that measures of the LRP provide a useful approach to test the predictions of the Adjusted Expectancy Model.

### Lateralized N200 related to response inhibition

The N200 amplitude increased with invalid as compared to valid response cues, predominately over the right hemisphere. This finding is consistent with previous findings which suggest that increased N200 amplitudes are related to the inhibition of a likely response [29-34]. The lateralization of the response cueing effect suggests that the processes related to response inhibition have been right lateralized in the present study. This finding corresponds to a recent literature review of data from neuroimaging studies and human lesion mapping which concludes that inhibitory processes are most often related to the right frontal cortex [35].

### Central N200 related to conflict processing

The model proposes that the activation of the cued response is increased in conditions with valid modality cues. This prediction was confirmed by measures of the LRP which showed that the activation of the cued response continued after target presentation when modality cues were valid rather than invalid irrespective of response cue validity. The N200 amplitude increased from the condition with two valid cues, over the condition with invalid modality and valid response cues, and conditions with two invalid cues. The largest N200 amplitude, however, was found with valid modality and invalid response cues. This pattern of results corresponds to the predictions derived from the Adjusted Expectancy Model.

Interestingly, the interaction of modality cueing and response cueing on the N200 amplitude did not vary with electrode position. Therefore, this interaction might reflect the operation of a more general control process possibly related to conflict-monitoring [36]. A conflict-monitoring interpretation of our finding is backed by the topography of the N200 amplitude effect and the fact that the largest N200 amplitude was found in the condition in which both the error rates and the incorrect response activation as measured by LRP amplitudes were maximal. The activation reflected in the N200 has been linked to a source in the medial frontal cortex [23,24,36]. The source of the N200 has been located in the anterior cingulate cortex (ACC) by dipole analyses [24] and a comparison of the variables that modulate both, ACC activity and the amplitude of the N200 [37]. According to a recent theory proposed by Carter and colleagues [24,38,39], the ACC is activated in response to conflict occurring between incompatible streams of information processing. This view accounts for numerous findings of increased ACC activity (and increased amplitudes of the N200) in conditions with increased response conflict (for a recent review see [39]).

In addition to the response cueing effects on the N200 amplitude, the present study showed significant modality cueing effects on the N200 amplitude. In conditions with valid response cues, the N200 amplitude was larger in trials with invalid than with valid modality cues. Because it seems unlikely that valid response cues induce a response conflict, this finding rather suggests that the N200 amplitude is also sensitive to perceptual sources of conflict. Numerous studies have provided evidence for the view that the N200 amplitude [24] and the ACC are sensitive to conflicts at the motor level. Only recently, findings from imaging studies suggested that there are additional sources of conflict. ACC activation was increased when conflict was induced by unexpected error feedback in a Wisconsin Card Sorting Task [40], and when subjects were reading stories that did not form an integrated narrative [41]. In addition, several studies found increased activation of ACC in response to perceptual conflict induced by the task-irrelevant global shape of a two-level stimulus in the global-local task [42], or by the irrelevant meaning of a word in the Stroop color-naming task [43]. Thus, the present finding of a modality cueing effect on the N200 amplitude contributes electrophysiological evidence to the view that the medial frontal cortex is generally activated in response to conflict occurring between incompatible streams of information processing [24,36,38,39].

## Conclusion

The present study examined the neurophysiology of combined expectancy effects when modality cueing was combined with response cueing. Expectancy effects on the LRP and the N200 amplitude are consistent with the predictions derived from the Adjusted Expectancy Model. When modality cues were valid, the activation of the expected response was increased leading to facilitated responses when the response cue was valid, but to increased conflict when the response cue was invalid. These findings support the view that expectancy effects which are related to relatively late levels of processing are adjusted online in response to the confirmation or disconfirmation of an expectancy related to relatively early levels of processing [9,10,12,18]. Analyses of the N200 amplitude suggest that different prefrontal processes contribute to this ERP component, including processes related to inhibition and to conflict-monitoring.

## Methods

### Subjects

Forty-four students from the University of Magdeburg participated in the experiment, 32 of them finished the screening session with less than 10 per cent errors, and 23 of this group showed expectancy interaction on measures of RT. The analysis of response-locked LRPs revealed an LRP in 18 subjects from this group (12 women, and 6 men) with 19 to 32 years of age (M = 23.1). The exclusion of trials with vertical eye-movements yielded a subgroup of 14 subjects for the N200 analyses. All subjects reported to be right-handed. All had normal or corrected-to-normal vision and none reported to have problems with color discrimination. All were naive as to the purposes of the experiment, and none had participated in a similar experiment before. Subjects took part in three 1-hr sessions and each received course credit or monetary compensation for participation. The study protocol had been approved by the local ethics committee.

### Task

In a two alternative choice reaction time task, subjects responded either to the color of a square, or to the pitch of a tone by pressing one of two keys with the index finger of their left and right hand. Pre-cues presented before the target stimulus indicated the likely stimulus modality of the target and the likely response required.

### Stimuli

Visual stimuli were presented in the center of the monitor as light-on-black images. Cues were the German words "Rot", "Grün", "Hoch", and "Tief" (Engl. red, green, high, and low). The word "red" ("low") cued the visual (auditory) modality as well as the response with the left hand. The word "green" ("high") cued the visual (auditory) modality as well as the response with the right hand (see Figure 2). Cues were presented in the center of the monitor for 1000 msec in white color subtending between 2° and 4° of visual angle. Visual targets were color squares subtending about 3.4° visual angle in red or green presented for 100 msec. Auditory targets were presented in double mono over two loudspeakers located 1° of visual angle on the left and right of the visual target. The speakers were hidden from view to prevent subjects from focusing their attention on a particular speaker. Tones of 1500 Hz or 300 Hz were presented for 100 msec. Error feedback was given by presenting the word "FALSCH" (Engl. wrong) subtending about 5.3° visual angle in yellow for 1000 ms.

### Design

A 2 × 2 repeated measures design was used with the two independent variables *modality cue *and *response cue*, each varying on two levels (valid vs. invalid). Dependent behavioral measures were RT and percentage errors. For every participant, a left (right) hand response was required by a red (green) signal or by a low (high) pitch. Each level of modality cue was combined with each level of response cue. Each target was preceded by each combination of modality cue and response cue, and all targets were equally frequent. Each cue was valid in 66 per cent of the trials and invalid in 33 per cent of the trials. Cue validity was realized by having more trials with valid cues than with invalid cues. Figure 2 gives the relative frequency of each of the four cue combinations for the example of a red target. The first session was considered practice, and the data of this session was not further analyzed. In the following two experimental sessions a practice block was followed by 12 Blocks of 60 trials in each session comprising 80 replications in each of the two most infrequent conditions with visual and auditory targets, respectively. Note that factors like target stimuli (red, green, and high, or low tone), session, and trial block varied orthogonally within the 2 × 2 design. However, the data were pooled across these factors to get a reliable estimate in conditions with small numbers of trials, and the analysis was restricted to the two relevant factors of this experiment – modality cue and response cue.

### Procedure

Subjects were tested individually in three sessions on separate days. They sat in a silent, sparsely illuminated room and viewed the computer monitor at a distance of about 80 cm. Subjects were informed about the validity of both aspects of the cue. They were instructed to keep their eyes on the fixation cross throughout the trial, to attend to and use the cues, and to respond to the target stimulus as quickly as possible without making too many errors. The sequence of events is given in Figure 3. Trials started with the cue presented for 1000 msec, followed by a fixation point for 1000 msec. Then the visual warning signal appeared for 500 msec and the target stimulus followed for 100 msec. Visual stimuli served as targets in half of the trials, auditory stimuli in the other half. Responses were given by pressing a response key with the left or right index finger. The computer monitored for a response within 1450 msec after target onset. In case of a wrong response, feedback was given after this period, followed by a rest of 1500 msec. The next trial started after 1000 msec.

### Data recording and analysis

During the accomplishment of the experimental task the electroencephalogram (EEG) was recorded from 30 electrodes including all 19 standard locations of the 10–20 System [44] with tin electrodes mounted in an elastic cap relative to a reference electrode placed on the left mastoid. Bipolar recordings of vertical and horizontal EOGs were made from sites above and below the left eye, and the right and left external canthi. Electrode impedance was maintained below 5 kOhm for all electrodes. The electrophysiological signals were amplified with a bandpass from 0.53 to 70 Hz with a digitization rate of 250 Hz using Schwarzer amplifiers and Brainlab recording software (O.S.G. bvba Brainlab, Rumst, Belgium). No digital filtering was applied to the signals.

#### LRP analyses

Stimulus-locked LRP waveforms were calculated from the EEG recordings at sites C3' and C4' in the epoch starting -748 msec prior to target onset and ending 1300 msec after target onset, after rejection of trials with artifacts. The average baseline activity in the epoch -748 msec to -648 msec prior to target onset was subtracted from every waveform. Note that the activity prior to cue onset was used as a baseline instead of the activity prior to target onset because the later waveforms were modulated by the cueing effects. Trials containing horizontal eye movements were removed (hEOG amplitude-range > 100 μV). To calculate LRPs, for each correct trial the activity on the side ipsilateral to the responding hand was subtracted from the activity contralateral to the side of the responding hand: i.e. for right hand responses C3' – C4', for left hand responses C4' – C3' was calculated [19,22]. These difference waveforms were then averaged over trials with left and right hand responses. A positive deflection of the LRP reflects activation of the incorrect response and a negative deflection activation of the correct response. Response-locked LRPs were similarly calculated in the epoch starting -1648 msec prior to the response and ending 400 msec after the response. For response-locked LRPs, the baseline was defined as the epoch -1648 msec to -1548 msec prior to the response. The effects on stimulus-locked LRP amplitudes were quantified as mean amplitude in three time intervals, -32 to 0 msec, 68–100 msec and 108–140 msec after target onset. LRP onset latencies were analyzed using a jackknife-based method [45] and F-values were corrected according to the proposal of Ulrich and Miller [46]: F_c _= F/(1-n)^2 ^with Fc denoting the corrected F-value and n the number of participants. As recommended by Miller et al. [45], the onset of stimulus locked LRPs was determined as the point in time where 50% of the LRP peak was exceeded, and in addition by using a fixed threshold of -1 μV. LRP-R intervals were determined by using a 90% threshold and the same fixed threshold. Analyses with the fixed 1 μV threshold are reported but the other analyses revealed a similar pattern of results.

#### N200 analyses

Regular stimulus-locked waveforms were also calculated from the EEG recordings at all electrode sites in the same epochs starting -748 msec prior to target onset and ending 1300 msec after target onset, after rejection of trials with artifacts. Again, the average baseline activity in the epoch -748 msec to -648 msec prior to target onset was subtracted from every waveform. For the regular ERPs, both trials containing horizontal and trials containing vertical eye movements were removed (EOG amplitude-range > 100 μV). We quantified the N200 effects after the application of a 3 Hz high-pass filter as the negative peak amplitude and latency in the time interval between 160 and 320 msec after target onset on the three frontal electrodes F3, Fz and F4. To examine the topography of the N200 effect in the condition with valid modality cues and invalid response cues (MvRi), we compared this condition to the condition with two valid cues (MvRv) by subtracting the MvRv condition from the MvRi condition. To assess whether the topography depends on the condition which is subtracted we repeated this calculation with the two other conditions MiRv and MiRi.

#### Statistical analysis

Choice RTs were summarized by means, determined for correct trials per subject and condition, excluding post-error trials [47]. RT and arc-sine transformed choice error rates as well as the ERP data were analyzed by a 2 × 2 repeated-measures analysis of variance (ANOVA) with factors modality cue and response cue. All reported p-values are based on Geisser-Greenhouse corrected degrees of freedom, while, for the sake of readability, the stated degrees of freedom are uncorrected.

## Authors' contributions

UM conceived and designed the study, accomplished the statistical analyses and drafted the manuscript. AVDL programmed the experiment, was responsible for the collection of the data, accomplished the analysis of the electrophysiological data and contributed to the manuscript. TFM contributed to the analyses and the manuscript.

## References

[B1] Spence C, Driver J (1997). On measuring selective attention to an expected sensory modality. Percept Psychophys.

[B2] Mattler U (2004). Combined expectancy effects are modulated by the relation between expectancy cues. Q J Exp Psychol.

[B3] Posner MI, Snyder CRR, Davidson BJ (1980). Attention and the detection of signals. J Exp Psychol: Gen.

[B4] Humphreys GW (1981). Flexibility of attention between stimulus dimensions. Percept Psychophys.

[B5] Posner MI, Snyder CRR, Solso R (1975). Attention and cognitive control. Information processing and cognition: The Loyola Symposium.

[B6] Neely JH (1977). Semantic priming and retrieval from lexical memory: Roles of inhibitionless spreading of activation and limited-capacity attention. J Exp Psychol: Gen.

[B7] Rosenbaum DA, Magill RA (1983). The movement precueing technique: Assumptions applications and extensions. Memory and control of action.

[B8] Rosenbaum DA, Kornblum S (1982). A priming method for investigating the selection of motor responses. Acta Psychol.

[B9] Kingstone A (1992). Combining Expectancies. Q J Exp Psychol.

[B10] Kingstone A, Klein R (1991). Combining shape and position expectancies: hierarchical processing and selective inhibition. J Exp Psychol: Hum Percept Perform.

[B11] Klein R, Hansen E (1987). Spotlight failure in covert orienting. Bull psychonomic society.

[B12] Klein R, Hansen E (1990). Chronometric analysis of apparent spotlight failure in endogenous visual orienting. J Exp Psychol: Hum Percept Perform.

[B13] Lambert A (1987). Expecting different categories at different locations and spatial selective attention. Q J Exp Psychol.

[B14] Lambert A, Hockey R (1986). Selective attention and performance with a multidimensional visual display. J Exp Psychol: Hum Percept Perform.

[B15] Klein R, Nickerson R (1980). Does oculomotor readiness mediate cognitive control of visual attention?. Attention and performance VIII.

[B16] Mattler U (2003). Combined perceptual or motor related expectancies modulated by type of cue. Percept Psychophys.

[B17] Handy TC, Green V, Klein RM, Mangun GR (2001). Combined expectancies: Event-related potentials reveal the early benefits of spatial attention that are obscured by reaction time measures. J Exp Psychol: Hum Percept Perform.

[B18] Mattler U (2005). Combined expectancy effects: An accumulator model. Cognitive Psychol.

[B19] Coles MGH (1989). Modern mind-brain reading: Psychophysiology, physiology and cognition. Psychophysiol.

[B20] Coles MGH, Gratton G, Donchin E (1988). Detecting early communication: Using measures of movement-related potentials to illuminate human information processing. Special Issue: Event related potential investigations of cognition. Biol Psychol.

[B21] Miller JO, Hackley SA (1992). Electrophysiological Evidence for Temporal Overlap Among Contingent Mental Processes. J Exp Psychol: Gen.

[B22] Leuthold H, Sommer W, Ulrich R (1996). Partial advance information and response preparation: Inferences from the lateralized readiness potential. J Exp Psychol: Gen.

[B23] Nieuwenhuis S, Yeung N, Cohen JD (2004). Stimulus modality perceptual overlap and the go/no-go N2. Psychophysiol.

[B24] Van Veen V, Carter CS (2002). The timing of action-monitoring processes in the anterior cingulate cortex. J Cognitive Neurosci.

[B25] Leuthold H, Jentzsch I (2002). Distinguishing neural sources of movement preparation and execution. An electrophysiological analysis. Biol Psychol.

[B26] Requin J, Brener J, Ring C, Jennings JR, Coles MGH (1991). Preparation for action. Handbook of Cognitive Psychophysiology.

[B27] Riehle A, Requin J (1993). The predictive value for performance speed of preparatory changes in neuronal activity of the monkey motor cortex and premotor cortex. Behaviroural Brain Res.

[B28] Osman A, Moore CM, Ulrich R (1995). Bisecting RT with lateralized readiness potentials: Precue effects after LRP onset. Acta Psychol.

[B29] Falkenstein M, Hoormann J, Hohnsbein J (1999). ERP components in Go/NoGo tasks and their relation to inhibition. Acta Psychol.

[B30] Kok A (1986). Effects of degradation of visual stimuli on components of the event-related potential (ERP) in go/nogo reaction tasks. Biol Psychol.

[B31] Kopp B, Mattler U, Goertz R, Rist F (1996). N2 P3 and the lateralized readiness potential in a nogo task involving selective response priming. Electroencephal Clin Neurophysiol.

[B32] Kopp B, Rist F, Mattler U (1996). N200 in the flanker task as a neurobehavioural tool for investigating executive control. Psychophysiol.

[B33] Pfefferbaum A, Ford JM, Weller BJ, Kopell BS (1985). ERPs to response production and inhibition. Electroencephal Clin Neurophysiol.

[B34] Sasaki K, Gemba H, Ono T, Squire LR, Raichle ME, Perrett DI, Fukuda M (1993). Prefrontal cortex in the organization and control of voluntary movement. Brain mechanisms of perception and memory: From neuron to behavior.

[B35] Aron AR, Robbins TW, Poldrack RA (2004). Inhibition and the right inferior frontal cortex. Trends Cogn Sci.

[B36] Ridderinkhof KR, Ullsperger M, Crone EA, Nieuwenhuis S (2004). The role of the medial frontal cortex in cognitive control. Science.

[B37] Van Veen V, Carter CS (2002). The anterior cingulate as a conflict monitor: fMRI and ERP studies. Physiol Behavior.

[B38] Botvinick MM, Nystrom LE, Fissell K, Carter CS, Cohen JD (1999). Conflict monitoring versus selection-for-action in anterior cingulated cortex. Nature.

[B39] Botvinick MM, Cohen JD, Carter CS (2004). Conflict monitoring and anterior cingulated cortex: an update. Trends Cogn Sci.

[B40] Monchi O, Petrides M, Petre V, Worsley K, Dagher A (2001). Wisconsin Card Sorting revisited: distinct neural circuits participating in different stages of the task identified by event-related functional magnetic resonance imaging. J Neuroscience.

[B41] Robertson DA, Gernsbacher MA, Guidotti SJ, Robertson RRW, Irwin W, Mock BJ, Campana ME (2000). Functional neuroanatomy of the cognitive process of mapping during discourse comprehension. Psychol Sci.

[B42] Weissman DH, Giesbrecht B, Song AW, Mangun GR, Woldorff MG (2003). Conflict monitoring in the human anterior cingulated cortex during selective attention to global and local object features. NeuroImage.

[B43] Milham MP, Banich MT, Barad V (2003). Competition for priority in processing increases prefrontal cortex's involvement in top-down control: an event-related fMRI study of the stroop task. Cognitive Brain Res.

[B44] Jasper HH (1958). The ten-twenty electrode system of the International Federation. Electroencephal Clin Neurophysiol.

[B45] Miller J, Patterson T, Ulrich R (1998). A Jackknife-based method for measuring LRP onset latency differences. Psychophysiol.

[B46] Ulrich R, Miller J (2001). Using the Jackknife-based scoring method for measuring LRP onset effects in factorial designs. Psychophysiol.

[B47] Rabbitt PMA (1966). Errors and error correction in choice-response tasks. J Exp Psychol.

